# A Predictive Model for Tumor Invasion of the Inferior Vena Cava Wall Using Multimodal Imaging in Patients with Renal Cell Carcinoma and Inferior Vena Cava Tumor Thrombus

**DOI:** 10.1155/2020/9530618

**Published:** 2020-10-06

**Authors:** Zhuo Liu, Liwei Li, Peng Hong, Guodong Zhu, Shiying Tang, Xun Zhao, Qiming Zhang, Guoliang Wang, Wei He, Hua Zhang, Heng Xue, Ligang Cui, Huiyu Ge, Jie Jiang, Shudong Zhang, Fangting Cao, Jing Yan, Fengrong Ma, Cheng Liu, Lulin Ma, Shumin Wang

**Affiliations:** ^1^Department of Urology, Peking University Third Hospital, Beijing, China; ^2^Department of Ultrasound, Peking University Third Hospital, Beijing, China; ^3^Department of Radiology, Peking University Third Hospital, Beijing, China; ^4^Research Center of Clinical Epidemiology, Peking University Third Hospital, Beijing, China

## Abstract

**Purpose:**

Developed a preoperative prediction model based on multimodality imaging to evaluate the probability of inferior vena cava (IVC) vascular wall invasion due to tumor infiltration.

**Materials and Methods:**

We retrospectively analyzed the clinical data of 110 patients with renal cell carcinoma (RCC) with level I-IV tumor thrombus who underwent radical nephrectomy and IVC thrombectomy between January 2014 and April 2019. The patients were categorized into two groups: 86 patients were used to establish the imaging model, and the data validation was conducted in 24 patients. We measured the imaging parameters and used logistic regression to evaluate the uni- and multivariable associations of the clinical and radiographic features of IVC resection and established an image prediction model to assess the probability of IVC vascular wall invasion.

**Results:**

In all of the patients, 46.5% (40/86) had IVC vascular wall invasion. The residual IVC blood flow (OR 0.170 [0.047-0.611]; *P* = 0.007), maximum coronal IVC diameter in mm (OR 1.203 [1.065-1.360]; *P* = 0.003), and presence of bland thrombus (OR 3.216 [0.870-11.887]; *P* = 0.080) were independent risk factors of IVC vascular wall invasion. We predicted vascular wall invasion if the probability was >42% as calculated by: {Ln [Pre/(1 − pre)] = 0.185 × maximum cornal IVC diameter + 1.168 × bland thrombus–1.770 × residual IVC blood flow–5.857}. To predict IVC vascular wall invasion, a rate of 76/86 (88.4%) was consistent with the actual treatment, and in the validation patients, 21/26 (80.8%) was consistent with the actual treatment.

**Conclusions:**

Our model of multimodal imaging associated with IVC vascular wall invasion may be used for preoperative evaluation and prediction of the probability of partial or segmental IVC resection.

## 1. Introduction

**Figure 1:**
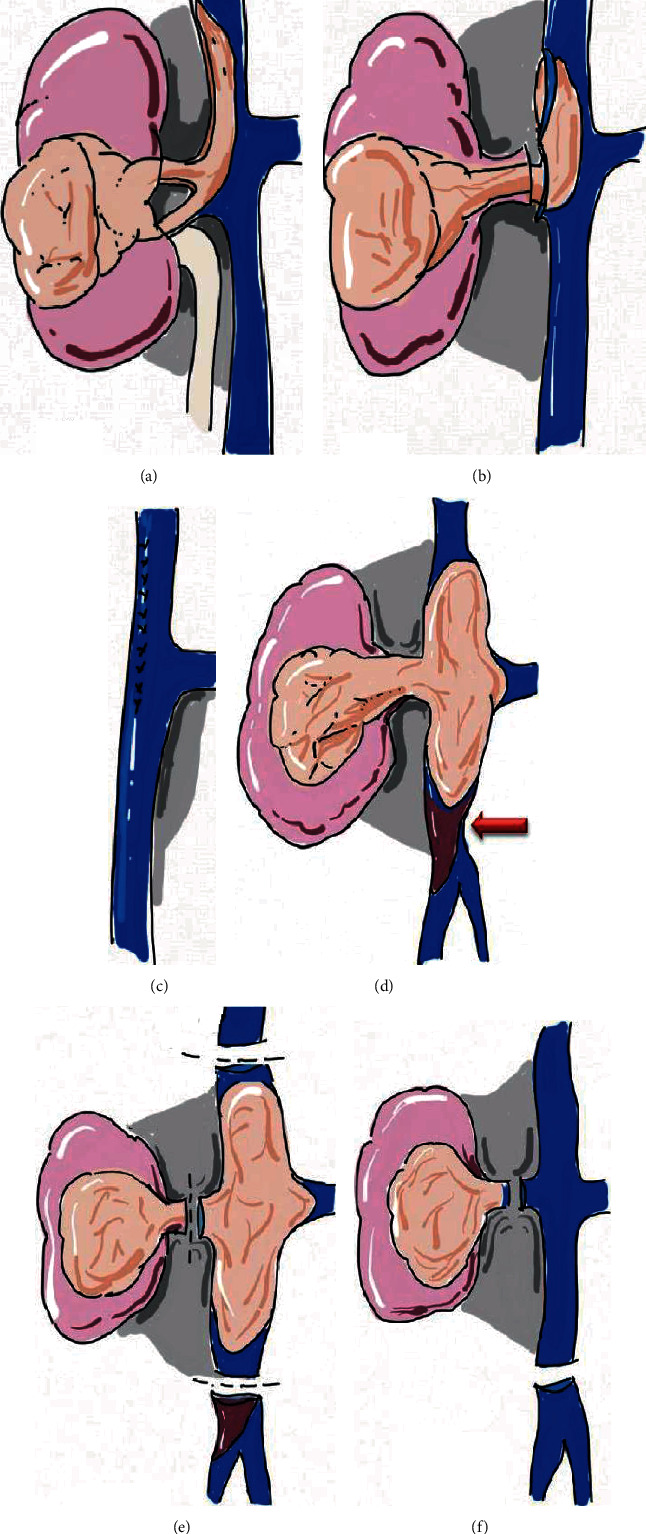
When tumor thrombus (TT) infiltrates into the inferior vena cava (IVC) vascular wall, IVC resection is necessary. (Figure 1(a)) partial resection the IVC involved by TT is needed (Figure 1(b)), and then suture of IVC. When the inferior vena cava tumor thrombus has completely obliterated the IVC and there is a simultaneous distal long bland thrombosis in the IVC (Figure 1(d), arrow), segmental resection (Figure 1(e)) of IVC (Figure 1(f)) should be performed.

Renal cell carcinoma (RCC) has a propensity for vascular growth [1] extending into the renal veins or inferior vena cava (IVC) in approximately 4-10% of patients [2–6]. Radical nephrectomy with thrombectomy may be a curative option [7–10]. However, thrombectomy is risky and technically challenging [6, 10–12]. Tumor invasion of the IVC wall is considered a risk factor for recurrence and poor prognosis in RCC. In order to achieve the purpose of radical resection of tumor, the invaded vascular wall needs to be removed [13]. According to the experience of resection IVC tumor thrombus (IVCTT) at our hospital, the influence of intraoperative IVC invasion on IVC resection is shown in Figure 1. It involves partial or segmental IVC resection, which requires vascular reconstruction using patch grafts or IVC resection and interruption. The management of IVC involving partial or segmental resection is critical for surgery. When a tumor thrombus (TT) infiltrates the IVC wall and adheres to the endothelium, partial resection is needed [14]. When the TT completely obliterates the IVC lumen and there is a distal long bland thrombosis in the IVC, segmental resection is recommended [13, 14]. Segmental resection often results in complex vascular reconstruction via vascular patches or interposition grafts [15]. Predicting IVC invasion preoperatively is advantageous for evaluating and planning surgical approaches and patient consultation.

The current RCC with IVCTT classification systems, such as the Mayo Clinic [16], Novick, and Hinman systems, only take into account the TT's location, which may be insufficient to intraoperatively evaluate the probability of IVC invasion [17]. Moreover, there is still no standard for classifying IVCTT on imaging [18]. Thus, IVC thrombectomy caused by IVC invasion often requires comprehensive preoperative imaging evaluation. At present, magnetic resonance imaging (MRI) is the first choice for diagnosing IVCTT [1, 19]. However, when the IVC blood flow decreases below a certain range due to tumor compression, the effect of slice saturation may lead to false-positive diagnoses [20]. Ultrasonography not only allows observation of the length and width of the IVCTT but also provides hemodynamic information, including the IVCTT and venal lumen blood flow in cases of IVC compression. Parameters from multiple imaging techniques could provide a more comprehensive tumor thrombus evaluation. Few studies have evaluated the potential factors predicting the probability of IVC resection by combining ultrasound with MRI/computed tomography (CT) findings.

Our objective was to screen for such predictors from MRI/CT and ultrasonic images and establish an image prediction model to preoperatively evaluate the probability of IVC invasion.

## 2. Patients and Methods

### 2.1. Patients

**Figure 2:**
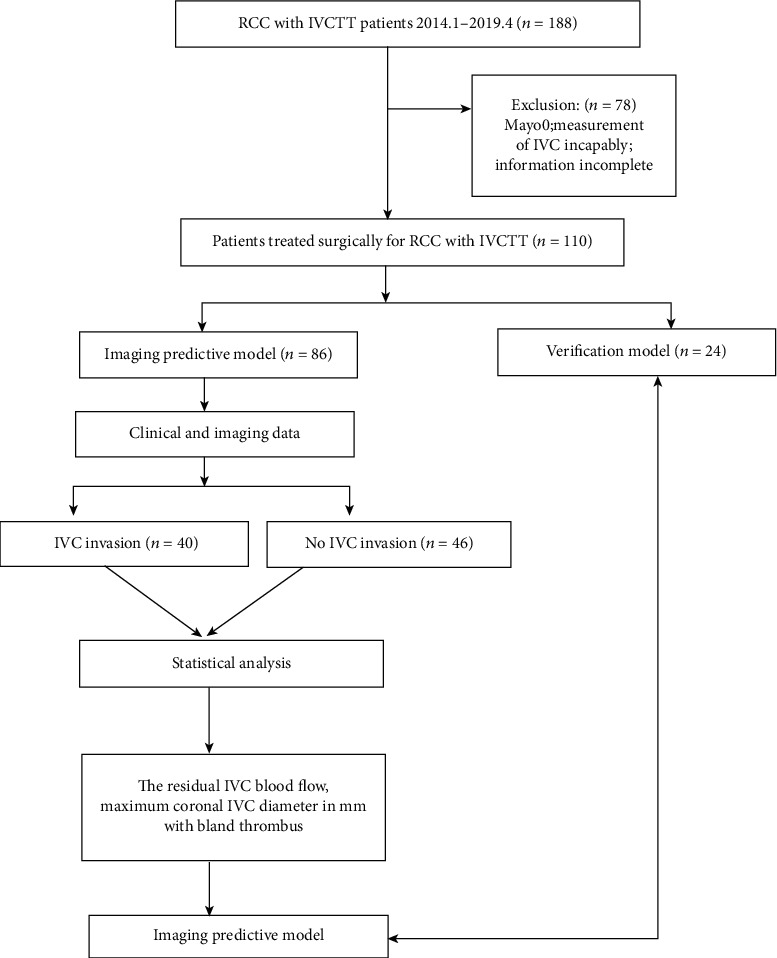
Summary of the study cohort and exclusion criteria.

We retrospectively reviewed 188 patients with RCC with IVCTT who underwent radical nephrectomy and IVC thrombectomy between January 2014 and April 2019. This study was approved by the institutional review board of the hospital involved (the number of the ethics approval: No.2018-396-01). All of the patients were preoperatively examined using ultrasound, CT, and/or MRI, and postoperative pathological examination was conducted to confirm RCC. Exclusion criteria included level 0 IVCTT (*n* = 49), incomplete imaging or pathology data (*n* =23), recurrent patients (*n* = 3), and the inability to measure IVC caused by IVC compression (*n* = 3). The remaining 110 patients with Mayo I-IV IVCTT formed this study's analytical cohort, 86 patients were used to establish the imaging model, and data validation was performed in 24 patients. The study cohort and exclusion criteria are listed in Figure 2.

### 2.2. Ultrasonography

Transabdominal ultrasonography and IVC ultrasound examination were conducted using a Convex array probe C1-6 MHz (LOGIQ E9, GE Healthcare, USA), C5-1 MHz (EPIQ 7, Philips Ultrasound, USA), C5-1 MHz (Hivision Ascendus, Hitachi, Japan), and CA1-7A MHz (RS80A, Samsung Medison, Korea). For level IV TT exceeding the diaphragm, cardiac ultrasound probes X5-1, S5-1 (EPIQ 7C, PHILIPS), and DM1-6A (RS80A, Samsung Medison, Korea) were used to observe the intra-atrial TT. All patients from 2014 to 2019 received routine ultrasound images. The patients were in the supine and lateral position to obtain clear images of the IVCTT. Two-dimensional, color, and spectrum Doppler was used to examine the IVCTT in detail. The length, echo, and color blood flow of the tumor embolus were recorded. All of the imaging data were archived in the ultrasound diagnostic department's database for subsequent analysis.

### 2.3. CT Scanning Sequence and Parameters

All of the patients underwent multidetector row CT (MDCT) (GE Revolution, GE Healthcare, USA) scanning using a CT and the energy spectrum scanning GSI Abd sequence. The scanner parameters in this study were as follows: 80-140 kV/190 mA; layer thickness, 5 mm; pitch, 0.99; rotation time, 0.8 s. The viewing parameters in this study were as follows: window width, 350 HU; window level, 40 HU; and matrix, 512 × 512. The upper boundary included the right atrium. Iopromide (iodine concentration: 370 mg/mL and 1.4 mL/kg) was injected through the anterior elbow vein at a rate of 4 mL/s. When the CT value of the ascending aorta was 120 HU, enhancement scanning was performed for 10 s (arterial phase), 35 s (venous phase), and 50 s (delayed phase).

### 2.4. MRI Examination Sequences and Parameters

All of the MRI examinations were conducted using a 3.0-T superconducting imaging system (Discovery MR750, GE Healthcare, USA) and abdominal array coils. The scanning scope included the upper and lower bounds of the IVCTT and the renal vein. The main MRI scanning parameters are listed in Table 1. Injection grade dimeglumine gadopentetate (20 mL and 20 mL/9.39 g) or gadoteric acid meglumine salt (15 mL and 377 mg/mL) were injected through the anterior elbow vein at a rate of 2-3 mL/s at a dosage depending on the patient weight (0.4 mL per kg of patient weight). Enhancement scanning was conducted for 20 s (arterial phase) and 40 s (venous phase).

### 2.5. Features of Patients with IVCTT

The clinical features included age, sex, RCC side, postoperative pathological diagnosis, IVCTT Mayo level, pathological type of RCC, and vascular wall invasion. Multimodal imaging analysis included both ultrasound and MRI/CT scans. The residual IVC blood flow was assessed via ultrasound. Two doctors specialized in ultrasound and blinded to the details of each patient's surgical procedure retrospectively analyzed the ultrasound blood flow images. Blood flow signals were labeled as 1 when present and 0 when no blood flow was indicated from the image.

The MRI/CT imaging data were evaluated by one radiologist blinded to the patients' surgery. The MRI/CT imaging parameters included maximum anterior-posterior (AP) IVC diameter, IVC AP diameter at the renal vein ostium (RVo), maximum coronal IVC diameter, coronal IVC diameter at the RVo, contralateral renal vein AP diameter at the RVo, maximum AP diameter of the renal vein, AP renal vein diameter, bland thrombus presence in the IVC, and complete IVC occlusion at the RVo.

### 2.6. Statistical Analysis

Shapiro Wilk (S-W) was used to test the normality of continuous variables. Continuous variables in accordance with normal distribution are summarized with mean ± standard deviation, and the two-sample independent *t*-test was used to analyze the clinical and imaging features of patients with or without IVC invasion. Nonparametric test analysis was applied to continuous variables that were not in accordance with normal distribution. Categorical features are summarized with frequency counts and percentages. For the categorical variables, comparisons were conducted using Pearson's chi-squared or Fisher's exact tests.

Univariable associations of the features with the probability of IVC invasion were evaluated using binary logistic regression to calculate the odds ratio (OR) and 95% confidence interval (CI). The multivariable model used stepwise selection by backward elimination (the Wald test), and the *P* value was 0.10, indicating the best feature selection. With sensitivity as the longitudinal axis and (1-specificity) as the horizontal axis, the variables obtained by multivariate analysis were drawn to evaluate the receiver operating characteristic curve (ROC) of IVC invasion. ROC enables us to choose a more optimal cutoff than 50%.

The kappa statistic was used to calculate the reproducibility of the assessments of the ultrasound doctors. Statistical analysis was conducted using SPSS 18.0 (IBM, Armonk, NY, USA). All of the tests were two-sided, and *P* values < 0.05 indicated statistical significance.

## 3. Results

Among the 86 patients, 46.5% (40/86) had IVC wall infiltration. Univariable associations of the clinical and radiographic features predicting vascular invasion during tumor thrombectomy are listed in Table 2. MRI and/or CT radiographic imaging showed that compared to the patients with no vascular wall invasion, factors that predicted the probability of IVC wall invasion were as follows: those with residual IVC vascular wall invasion were significantly more likely to have distal bland thrombosis (*P* < 0.001), complete IVC occlusion at the RVo (*P* < 0.001), and no residual blood flow (*P* < 0.001) and a significantly larger maximum AP IVC diameter 34.1 (29.2, 40.4) mm vs. 26.4 (22.1, 32.0) mm, *P* < 0.001; IVC AP diameter at the RVo 30.1 (25.6, 34.4) mm vs. 24.1 (22.3, 28.9) mm, *P* < 0.001; and a maximum coronal IVC diameter (36.3 ± 5.6) mm vs. (29.2 ± 4.8) mm, *P* < 0.001.

**Figure 3:**
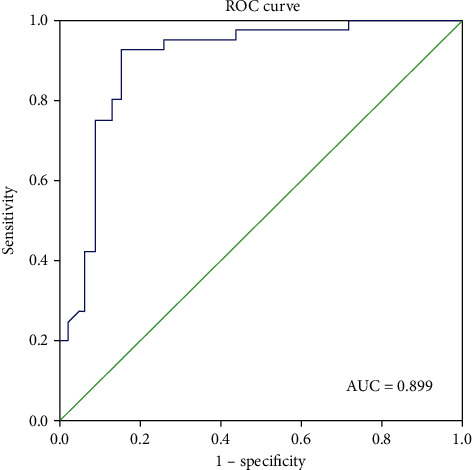
Receiver operating characteristic (ROC) curve analysis of predicted probabilities of invasion of inferior vena cava (IVC) wall invasion according to the combined with the three features: maximum coronal IVC diameter; residual IVC blood flow on ultrasound and with bland thrombus. Area under curve (AUC) of ROC is 0.899 [0.829-0.969].

In the final multivariable model (Table 3), three features were used to predict the probability of IVC wall invasion: the residual IVC blood flow (OR 0.170 [0.047-0.611]; *P* = 0.007), maximum coronal IVC diameter in mm (OR 1.203 [1.065-1.360]; *P* = 0.003), and presence of bland thrombus (OR 3.216 [0.870-11.887]; *P* = 0.080). Combining these three features, we found that the area under the curve (AUC) of the receiver operating characteristic (ROC) was 0.899 [0.829-0.969] (Figure 3). According to the ROC curve, we found that the cut-off value of 0.42 is better.

**Figure 4:**
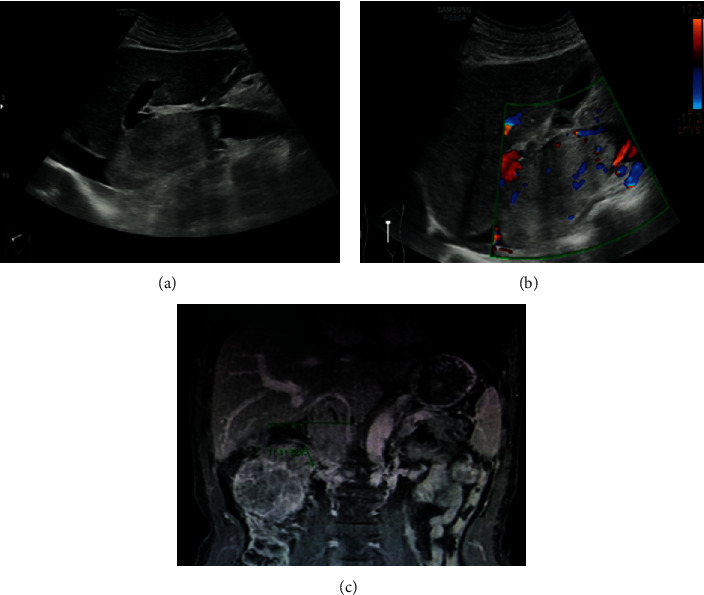
Imaging and pathological findings in a 63-year-old male with clear cell renal cell carcinoma (ccRCC) in the right kidney with inferior vena cava (IVC) tumor thrombus (IVCTT, Mayo III). (a) Ultrasound images showing that the IVC is completely obstructed and has no residual blood flow. (b) Coronal gadolinium enhanced T1-weighted image showing the IVCTT and its maximum coronal diameter of 37.5 mm. The possibility of IVC invasion was 68% in this case. In consideration of tumor invasion of IVC wall, so IVC resection was performed.

The final model to predict the probability of IVC invasion is summarized as follows (Table 4):

(1) $$\begin{array}{c} \text{Ln}\left\{\frac{\text{pre}}{1-\text{pre}}\right\}=0.185\times \text{maximum cornal IVC diameter}+1.168\times \text{bland thrumbus}-1.770\times \text{residual IVC blood flow}-5.857, \end{array}$$

where the residual IVC blood flow variable is 1 or 0 and “pre” is the probability of IVC vascular wall invasion. We calculated the probability of IVC invasion (Figure 4). A probability ≤42% indicated no IVC vessel wall invasion. A probability >42% indicated IVC vessel wall invasion. Among all of the patients, the predicted invasion probability was consistent with the actual operation in 76/86 (88.4%) but opposite in 11.6% (10/86) of the cases. The false-positive rate was 8.1% (7/86), and the false-negative rate was 3.5% (3/86).

In the literature, a strong interobserver concordance and reproducibility of measurements of vascular diameters on MRI scans were confirmed, ranging from 0.93 to 0.99 [20]. In this study, we assessed the concordance correlation coefficient for estimating blood flow in the residual lumen using two sonographers. The kappa of the blood flow in the residual lumen was 0.77; therefore, the diagnostic results provided by the ultrasound doctors were consistent.

## 4. Discussion

RCC is a common malignant tumor of the urinary system. IVCTT occurs in 4-10% of RCC cases [20]. Radical nephrectomy and IVC thrombectomy can effectively improve the prognosis of locally advanced RCC, with a disease-specific survival rate of 40% to 60% at 5 years [21, 22]. A previous study reported that 6-8% of patients undergoing tumor thrombectomy required segmental IVC excision [13]. The objective of surgical treatment for RCC with IVCTT is to completely remove all tumor burden. Therefore, it is necessary to remove the vascular wall infiltrated by IVCTT. However, IVC infiltrated by IVCTT is a risk factor for poor prognosis [23] and an important factor for IVC resection. Thus, a comprehensive preoperative imaging evaluation may allow surgical urologists to more effectively treat the IVC [19, 21].

Our data showed that 46.5% (40/86) of the patients had IVC invasion. Using univariate and multivariate analysis, we identified three strong indicators and significant risk factors for IVC invasion: the coronal maximum IVC diameter in MRI/CT, the absence of color flow signal on ultrasound, and the presence bland thrombus.

Previous research on IVCTT suggested that the most tolerable invasion area of the IVC wall is the RVo [13]. The IVC diameter at the RVo can be used to predict IVC resection and invasion of the IVC wall. Psutka et al. [13] indicated that previous literature had not reported the endpoint for the probability of IVC resection or potential complex IVC reconstruction caused by IVC invasion. Their research also showed that Mayo Clinic risk factors on radiologic findings consisted of three features. Overholser et al. [24] reported that these factors could not predict IVC vascular invasion and reconstruction. Similar to these studies, we found that the maximum coronal IVC diameter is a risk factor for IVC resection. The data showed that, in 82.6% (71/86) of the cases, this diameter was the same as the coronal diameter of the renal vein at the RVo, which was closer to the proximal part of the IVC. However, the two diameters were different in 17.4% (15/86) of the cases. This may have been related to the kinetics of tumor invasion and metastasis. Cancer cells invade tissues through two mechanisms: growth-related tumor expansion and cancer cell locomotion. Tumor growth generates expansive forces that tend to push the tumor along paths of least resistance [25]. A total of 80% (12/15) were classified as WHO/ISUP 2016 nuclear grade III-IV. This might explain why, in some of the patients, the maximum coronal IVC diameter was different from that of the renal vein at the RVo that was closer to the proximal part. The underlying mechanism needs further investigation; nevertheless, our results suggest that attention should be paid not only to the IVC at the RVo but also to its maximum coronal diameter.

Another imaging parameter of the predictive model was the residual IVC blood flow on ultrasound, which is a protective factor for the invasion of the IVC wall. Psutka et al. [13] reported that one of the risk factors for IVC resection was complete obstruction of the IVC at the RVo. The residual IVC blood flow on ultrasound was detected, which indicated that there was a space in the IVC and the lumen of the IVC had not been completely obstructed by tumor thrombus. Besides, we posit that the flow and scouring of the residual IVC blood flow can prevent cancer cells from adhering to the vascular wall, hindering the formation of tumor thrombus.

Previous studies focused on IVC using MRI/CT images, but there is limited research on the evaluation of IVCTT via ultrasound. It is difficult to assess the presence of IVCTT by MRI when the IVC is compressed by large tumors and enlarged lymph nodes. False-positive MRI results can occur when the IVC blood flow decreases, the flow velocity decelerates, and the saturation effect is obvious [26].

The advantage of ultrasound is that it can display blood flow signals dynamically in real time. Ultrasound can detect blood flow signals in the residual lumen, which indirectly proves that the IVC lumen is not completely obstructed. By adjusting the parameters, ultrasound can display the low-velocity blood flow well and remedy the misdiagnosis of MRI/CT caused by the pressure of the lumen and the decrease in the flow velocity, increasing the accuracy of evaluating IVC invasion.

The third predictor of IVC invasion was bland thrombus distal of the IVC. Catalano et al. [27] retrospectively analyzed the causes of bland thrombus in patients with cancer because of the Virchow triad in cancer patients such as endothelial injury, stasis of flow caused by vascular infiltration by tumor mass or blood hyperviscosity, and activation of clotting. In patients with RCC and IVCTT, bland thrombus is often accompanied by tumor thrombus, especially locally progressive RCC. Hutchinson et al. [14] analyzed 446 patients with radical nephrectomy with thrombectomy at five medical centers to study the effect of bland thrombus (mainly composed of activated platelets, macrophages, and fibrin) on their survival and the increase in cancer-specific mortality (CSM) in patients with thrombosis. Studies on RCC have shown that immunoreactive RCC can cause aggressive tumor behavior and enhanced immune surveillance. Immune surveillance may lead to hypercoagulability, which may easily form bland thrombus [14].

The influence of bland thrombus on surgery is related to the establishment of collateral circulation and the length of bland thrombus, which decides the IVC management. Overall, 50% of patients with bland thrombus require IVC segmental resection or partial resection, potentially challenging for surgeons.

We developed a multivariable model to predict the probability of IVC invasion combining the maximum coronal IVC diameter and residual IVC blood flow on ultrasound and with bland thrombus. The AUC of the ROC curve was 0.899 [0.829-0.969], so the model exhibited significant predictive value. The predicted resection probability was consistent with the actual surgery in 76/86 (88.4%) of the patients. Thus, this model may be used for preoperative planning and individually calculating the resection probability of each patient.

In our previous research, we found that increase in maximum AP diameter of venous tumor thrombus diameter at the renal vein ostium (RVo) and complete occlusion of the IVC are independent risk factors for a higher probability of IVC wall invasion by tumor thrombus. The probability of intraoperative IVC resection for a patient with the absence of both independent factors, AP diameter of the VTT at the RVo greater than 17.0 mm, IVC occlusion, or the two concurrent factors is 4.5%, 22.7%, 55.5%, and 65.7%, respectively. Based on previous research, this study developed the following contents: firstly, we designed a prediction model that evaluated the potential factors predicting the probability of IVC resection by combining ultrasound with MRI/CT findings. Ultrasonography not only allows observation of the length and width of the IVCTT but also provides hemodynamic information, including venal lumen blood flow in cases of IVC compression. Parameters from multiple imaging techniques could provide a more comprehensive tumor thrombus evaluation. Secondly, we found that bland thrombus was often accompanied by tumor thrombus, especially locally progressive RCC. Overall, 50% of patients with bland thrombus required IVC segmental resection or partial resection. We identified the maximum coronal IVC diameter in MRI/CT, color flow signal absence on ultrasound, and the presence of bland thrombus as three key parameters of multimodal imaging associated with IVC invasion. In the validation patients, 21/24 (80.8%) was consistent with the actual treatment.

This study had some limitations due to its retrospective nature, leading to a potential selection bias. Moreover, the reference standard of wall invasion was used as the intraoperative finding. In addition, it used a single institution's clinical experience and practice patterns; thus, a multicenter validation is necessary. In the future, more cases should be examined to verify our prediction model.

## 5. Conclusions

We identified the maximum coronal IVC diameter in MRI/CT, color flow signal absence on ultrasound, and the presence of bland thrombus as three key parameters of multimodal imaging associated with IVC invasion. Our prediction model can be used during preoperative planning to evaluate and predict the probability of partial or segmental IVC resection.

## Figures and Tables

**Table 1:** Sequence parameters for IVCTT with abdominal array coils at 3.0-T superconducting imaging system.

	Imaging plane	Freq FOV^a^	Phase-FOV	TR/TE^b^ (ms)	Slice thickness/spacing (mm)	Band width (kHz)	Flip angle (°)	NEX^c^	Time
T2 FS RTr propeller ^d^	Axial		36	9000/82	6.0/1.0	62.5			2 min
T2 SSFSE^e^	Sagittal	40	0.7	1028/70	6.0/1.0	62.5			18 s BH^f^
	Coronal	40	0.9	1216/70	6.0/1.0	62.5			22 s
LAVA ^g^-Flex	Axial	40	0.8	4.0/1.8	5.0	142.86			20 s BH
LAVA-Flex 3	Axial	36	0.8	4.0/1.8	5.0	142.86	10	1.0	1 min
LAVA-Flex +C	Sagittal	35	1	4.4/1.9	3.0	142.86	15	1.0	23 s BH
	Coronal								24 s BH
DWI ^h^	Axial	35	1	4800/62	6.0/1.0	250			3 min, 54 s

^a^FOV: field-of-view; ^b^TR: repetition time TE: echo time; ^c^NEX: number of excitations; ^d^FS: fat suppression; RTr: Respiratory trigger; ^e^SSFSE: Single Shot Fast Spine Echo; ^f^BH: breath-hold; ^g^LAVA: Liver Acquisition with Volume Acceleration; ^h^DWI: diffusion-weighted imaging.

**Table 2:** Univariable associations of multimodal image parameters predicting vascular wall invasion.

Features	IVC invasion		*Z*/*t*/*χ*^2^	*P*
	Yes (*n* = 40)	No (*n* = 46)		
	Median (IQR)			
Age, *y*	60.0 (52.3, 63.8)	61.0 (53.3, 66.0)	-0.485	0.627
AP diameter renal vein	30.1 (25.6, 34.4)	24.1 (21.3, 28.9)	-0.102	0.271
Maximum IVC AP diameter, mm	34.1 (29.2, 40.4)	26.4 (22.1, 32.0)	-3.824	<0.001
IVC AP diameter at the RVo, mm	30.1 (25.6, 34.4)	24.1 (22.3, 28.9)	-3.887	<0.001
Contralateral renal vein AP diameter at the RVo, mm	10.4 (8.3, 12.1)	9.1 (7.9,10.9)	-1.677	0.930
	$\bar{x}\pm s$			
Maximum coronal IVC diameter, mm	36.3 ± 5.6	29.2 ± 4.8	2.117	<0.001
Coronal IVC diameter at the RVo, mm	35.2 ± 5.5	28.7 ± 5.0	0.830	<0.001
Maximum AP diameter of renal vein	21.9 ± 5.2	21.7 ± 5.6	0293	0.867
		*n* (%)		
Sex			3.958	0.047
Male	30 (75.0)	25 (54.3)		
Female	10 (25.0)	21 (45.7)		
Side of tumor			0.006	1.000
Right	29 (72.5)	33 (71.7)		
Left	11 (27.5)	13 (28.3)		
Tumor thrombus level			10.471	0.015
Mayo I	3 (7.5)	14 (30.4)		
Mayo II	16 (40.0)	20 (43.6)		
Mayo III	14 (35.0)	6 (13)		
Mayo IV	7 (17.5)	6 (13)		
Pathological type			2.036	0.361
Clear cell renal cell carcinoma	28 (70.0)	38 (82.6)		
Papillary renal cell carcinoma	7 (17.5)	4 (8.7)		
Other	5 (12.5)	4 (8.7)		
Vascular wall invasion by pathological examination			5.070	0.031
No	26 (65.0)	39 (84.8)		
Yes	12 (30.0)	5 (10.9)		
Unsure	2 (5.0)	2 (4.3)		
Bland thrombus in the IVC on CT/MRI			19.822	<0.001
No	15 (62.5)	39 (84.8)		
Yes	25 (37.5)	7 (15.2)		
Complete IVC occlusion at the RVo on CT/MRI			12.825	<0.001
No	5 (12.5)	21 (45.7)		
Yes	34 (80.5)	21 (45.7)		
Unsure	1 (2.5)	4 (8.6)		
Residual IVC blood flow on ultrasound			19.873	<0.001
No	33 (82.5)	16 (34.8)		
Yes	7 (17.5)	30 (65.2)		

IVC: inferior vena cava; AP: anterior-posterior; IVCTT: IVC tumor thrombus; RVo: renal vein ostium; CT/MRI: computed tomography/magnetic resonance imaging; *P* < 0.05 indicates statistical significance.

**Table 3:** Multivariable associations of multimodal image parameters predicting IVC wall invasion.

Features	Univariate analysis OR (95% CI)		Multivariable analysis OR (95% CI)	*P*
Maximum IVC AP diameter, mm	6.580 (3.295-9.865)	<0.001		
IVC AP diameter at the RVo, mm	1.181 (1.081-1.290)	<0.001		
Residual IVC blood flow	0.477 (0.288-0.666)	<0.001	0.170 (0.047-0.611)	0.007
Maximum coronal IVC diameter, mm	7.101 (4.887-9.314)	<0.001	1.203 (1.065-1.360)	0.003
Coronal IVC diameter at the RVo, mm	6.489 (4.219-8.753)	<0.001		
Bland thrombus in the IVC on CT/MRI	0.473 (0.289-0.656)	<0.001	3.216 (0.870-11.887)	0.080
Complete IVC occlusion at the RVo on CT/MRI	0.372 (0.180-0.564)	<0.001		

OR: odds ratio; CI: confidence interval; AP: anterior-posterior; IVC: inferior vena cava; IVCTT: IVC tumor thrombus; RVo: renal vein ostium; CT/MRI: computed tomography/magnetic resonance imaging.

**Table 4:** Multivariable model to predict IVC wall invasion.

	B	OR (95% CI)	*P*
Residual IVC blood flow	-1.770	0.170 (0.047-0.611)	0.007
Maximum coronal IVC diameter	0.185	1.203 (1.065-1.360)	0.003
Bland thrombus	1.168	3.216 (0.870-11.887)	0.080
Intercept	-5.857	0.000	

IVC: inferior vena cava; B: regression coefficient; OR: odds ratio; CI: confidence interval.

## Data Availability

The datasets analyzed during the current study available from the corresponding author on reasonable request.
